# ASSESSING THE NEEDS OF OLDER ADULTS IN RURAL WEST TEXAS

**DOI:** 10.1093/geroni/igad104.2860

**Published:** 2023-12-21

**Authors:** Suzie Macaluso, Julia Free

**Affiliations:** Abilene Christian University, Abilene, Texas, United States; Abilene Christian University, Abilene, Texas, United States

## Abstract

With 1 in 5 adults over the age of 65 living in rural areas, understanding their unique concerns and needs becomes increasingly important. This study was carried out in partnership with the West Central Texas Area Agency on Aging (WCT AAA) to identify the top concerns of older adults in rural West Texas. The survey was conducted in person at four locations across the region served by the WCT AAA. A total of 183 respondents completed the survey and their responses to the question “what are the biggest issues facing older adults today” along with demographic information were collected. Participants were between the ages of 56 and 95 years old with a mean age of 76 years old. 60% of the participants were female and 40% were male. Respondents were predominantly White, 80%, with 16% Hispanic or Latino, and 3% Black or African American. The most common concern among respondents was physical health and well being with the increasing cost of living being the second most frequent response. Additionally, residents from more rural counties reported social interaction as more important than either health or cost of living. These findings highlight the need for targeted interventions to address the concerns of older adults in rural areas, particularly related to healthcare access and social isolation. Strategies such as telemedicine and community-based programs to address transportation and social isolation may be particularly effective in addressing these concerns.

